# An Integrated Influenza Surveillance Framework Based on National Influenza-Like Illness Incidence and Multiple Hospital Electronic Medical Records for Early Prediction of Influenza Epidemics: Design and Evaluation

**DOI:** 10.2196/12341

**Published:** 2019-02-01

**Authors:** Cheng-Yi Yang, Ray-Jade Chen, Wan-Lin Chou, Yuarn-Jang Lee, Yu-Sheng Lo

**Affiliations:** 1 Graduate Institute of Biomedical Informatics Taipei Medical University Taipei Taiwan; 2 Department of Surgery School of Medicine College of Medicine, Taipei Medical University Taipei Taiwan; 3 Taipei Medical University Hospital Taipei Taiwan; 4 Division of Infectious Disease Department of Internal Medicine Taipei Medical University Hospital Taipei Taiwan

**Keywords:** influenza, epidemics, influenza surveillance, electronic disease surveillance, electronic medical records, electronic health records, public health

## Abstract

**Background:**

Influenza is a leading cause of death worldwide and contributes to heavy economic losses to individuals and communities. Therefore, the early prediction of and interventions against influenza epidemics are crucial to reduce mortality and morbidity because of this disease. Similar to other countries, the Taiwan Centers for Disease Control and Prevention (TWCDC) has implemented influenza surveillance and reporting systems, which primarily rely on influenza-like illness (ILI) data reported by health care providers, for the early prediction of influenza epidemics. However, these surveillance and reporting systems show at least a 2-week delay in prediction, indicating the need for improvement.

**Objective:**

We aimed to integrate the TWCDC ILI data with electronic medical records (EMRs) of multiple hospitals in Taiwan. Our ultimate goal was to develop a national influenza trend prediction and reporting tool more accurate and efficient than the current influenza surveillance and reporting systems.

**Methods:**

First, the influenza expertise team at Taipei Medical University Health Care System (TMUHcS) identified surveillance variables relevant to the prediction of influenza epidemics. Second, we developed a framework for integrating the EMRs of multiple hospitals with the ILI data from the TWCDC website to proactively provide results of influenza epidemic monitoring to hospital infection control practitioners. Third, using the TWCDC ILI data as the gold standard for influenza reporting, we calculated Pearson correlation coefficients to measure the strength of the linear relationship between TMUHcS EMRs and regional and national TWCDC ILI data for 2 weekly time series datasets. Finally, we used the Moving Epidemic Method analyses to evaluate each surveillance variable for its predictive power for influenza epidemics.

**Results:**

Using this framework, we collected the EMRs and TWCDC ILI data of the past 3 influenza seasons (October 2014 to September 2017). On the basis of the EMRs of multiple hospitals, 3 surveillance variables, TMUHcS-ILI, TMUHcS-rapid influenza laboratory tests with positive results (RITP), and TMUHcS-influenza medication use (IMU), which reflected patients with ILI, those with positive results from rapid influenza diagnostic tests, and those treated with antiviral drugs, respectively, showed strong correlations with the TWCDC regional and national ILI data (*r*=.86-.98). The 2 surveillance variables—TMUHcS-RITP and TMUHcS-IMU—showed predictive power for influenza epidemics 3 to 4 weeks before the increase noted in the TWCDC ILI reports.

**Conclusions:**

Our framework periodically integrated and compared surveillance data from multiple hospitals and the TWCDC website to maintain a certain prediction quality and proactively provide monitored results. Our results can be extended to other infectious diseases, mitigating the time and effort required for data collection and analysis. Furthermore, this approach may be developed as a cost-effective electronic surveillance tool for the early and accurate prediction of epidemics of influenza and other infectious diseases in densely populated regions and nations.

## Introduction

Influenza is a contagious respiratory illness caused by influenza viruses, which are easily transmitted from person to person during seasonal epidemics. Infection can result in severe illness, hospitalization, and even death, particularly among the elderly, very young individuals, and individuals with preexisting medical conditions [1,2]. According to the World Health Organization (WHO), an estimated 3 to 5 million cases of seasonal influenza epidemics develop severe illness, resulting in 250,000 to 500,000 annual deaths worldwide [3]. Moreover, influenza contributes to heavy economic losses to individuals and communities [4,5]. Therefore, early detection and treatment are the top priorities of health care professionals to reduce mortality and morbidity because of influenza.

Several surveillance methods for the real-time detection and routine monitoring of influenza activity have been proposed. Traditional influenza surveillance systems primarily rely on influenza-like illness (ILI) and virology data reported by health care providers, including hospitals, clinics, and contract laboratories [6,7]. The most notable are the official Centers for Disease Control and Prevention (CDC) reports on influenza activity in the United States and other countries, which despite their high detection accuracy, typically have a time lag of 1 to 2 weeks from incidence to report completion [8-10]. Therefore, simple, accurate, and timely surveillance approaches may be beneficial for the society.

Nontraditional influenza surveillance systems have attempted to provide more timely estimates of influenza activity using alternative or novel data sources. Compared with traditional approaches, nontraditional surveillance systems are more often aimed at the real-time detection of influenza epidemics. Some use sentinel-based surveillance systems with alternative data sources, such as flu prescription drug sales and school absenteeism data, to estimate the total number of patients with influenza [11-13]. These sentinel-based surveillance systems often have limitations, such as participating rates in the official sentinel surveillance, lack of influenza-specific data, and potential overestimation of the number of patients with influenza [13-15]. Some systems attempt to use novel data sources from the internet, such as Google user search logs and social media stream data, to capture influenza trends in near real time [16-19]; however, such strategies capture user behaviors, thoughts, and subjective judgments, as reflected through keywords, without physician confirmation. Unfortunately, these surveillance systems remain plagued by limitations in the accuracy, difficulties in recognizing influenza outbreaks at local and regional scales, and lack of support by clinical evidence and are often viewed as an extension of traditional surveillance systems [20-25]. Other approaches might use hospital-based data—electronic health records, emergency department (ED) patients’ diagnosis codes or chief complaints, or rapid influenza diagnostic tests (RIDTs) results—to conduct near-real-time influenza surveillance and detect local and regional outbreaks. The results of such approaches are considered to be complementary to the regular reports of the national CDC [26-29]. Therefore, real-time and accurate monitoring, early prediction, and control of influenza epidemics remain major global public health challenges [16,30,31].

In Taiwan, seasonal influenza epidemics occur in winter, from late November to March, although smaller epidemics may occur during summer. The influenza surveillance and reporting system of Taiwan CDC (TWCDC) is centrally organized and aided by the regional departments of health, contracted laboratories, and all health care institutes. The Taiwan National Infectious Disease Statistics System [32] reports information regarding influenza activity, such as (1) viral surveillance data, including the percentage of positive specimens, viral types, trends, antigenicity, and antiviral resistance; (2) ILI surveillance data, including proportions of outpatient department (OPD) and emergency department visits attributable to ILI; and (3) reports of severe complicated influenza and mortality surveillance data. The TWCDC ILI reports, including national and regional ILI data, are freely available on the website but show a similar delay of at least one week for studies of OPD and ED visits for ILI and 2 weeks for studies of virology [32]. This information represents the gold standard for clinical and epidemiological studies of influenza epidemics in Taiwan. Over the past 3 years (October 2014 to September 2017), TWCDC has reported 2 influenza epidemics in Taiwan, 1 from the winter of 2015 until the spring of 2016 and another during the summer of 2017.

In 1995, Taiwan introduced a government-administered, insurance-based National Health Insurance (NHI) system. This single-payer NHI system of Taiwan comprehensively covers the population and has good accessibility, short waiting times, and relatively low costs. However, the weaknesses of this type of health care system include the variable quality of care, weak gatekeeper role, and increasing financial pressures [33,34]. Therefore, patients are free to seek clinical services without referral when they feel uncomfortable. Consequently, the first-line physicians in OPDs or EDs may be in the best position to detect influenza activity early. Most patients with positive RIDT results are treated with antiviral drugs. RIDTs take only 15 to 20 min to obtain results, but they vary in sensitivity and specificity when compared with the other methods, such as viral culture or reverse transcription polymerase chain reaction (RT-PCR). In particular, RIDTs have high specificities (90%-95%), but their sensitivities are approximately 50% to 70% [35]. However, because of the long examination time (ie, several hours to days to obtain test results), specific testing equipment requirements, and higher costs of viral cultures or RT-PCR, most health care facilities tend to consider these as second-line diagnostic tools while mainly relying on RIDTs for influenza. Furthermore, because of low sensitivities of RIDTs, administration of antiviral drugs is suggested for individuals with ILI as well as for those presenting with other risk factors, such as underlying diseases, immunosuppression, or family environments with the potential for cluster infections in the absence of positive RIDT results.

When a patient is suspected or confirmed to have a diagnosis of influenza, most of the data on clinical history, laboratory results, received medications, and diagnostic codes are electronically documented in the hospital’s electronic medical records (EMRs). Therefore, using EMR systems, patients with ILI, patients with positive RIDTs, and patients prescribed antiviral drugs can be easily identified, thereby allowing timely monitoring of influenza trends. Furthermore, by scaling up EMRs of a single hospital to a regional health care system, influenza epidemics may be predicted more accurately. Therefore, the use of selected surveillance data from hospital-based EMRs may be considered to aid the early prediction of regional and national influenza epidemics [36].

In this study, we aimed to develop a framework for integrating EMRs of multiple hospitals and the TWCDC ILI data, with the goal of reducing the time and effort involved in data collection and analysis for influenza surveillance. To improve regional and national influenza surveillance and control, we identified surveillance variables on the basis of physician behaviors for the early prediction of influenza epidemics and evaluated the predictive ability of each variable over the study period.

## Methods

### Settings

This study was conducted in the Taipei Medical University Health Care System (TMUHcS), which operates 3 hospitals: Taipei Medical University Hospital, Wan Fang Hospital, and Shuang Ho Hospital. All 3 hospitals are located in the Taipei metropolitan area (Taipei City and New Taipei City), within an 11-km driving distance from each other, and are Joint Commission International-certified academic research centers providing tertiary care. The EMRs of the 3 hospitals are hosted on a secure and private network for easy accessibility and interoperability. The total capacity of the 3 hospitals is 2800 beds, and they receive over 300,000 OPD visits each month. On an average, clinical reimbursement of TMUHcS accounts for 3.2% of the total NHI budget.

### Integrated Influenza Surveillance Framework

**Figure 1:**
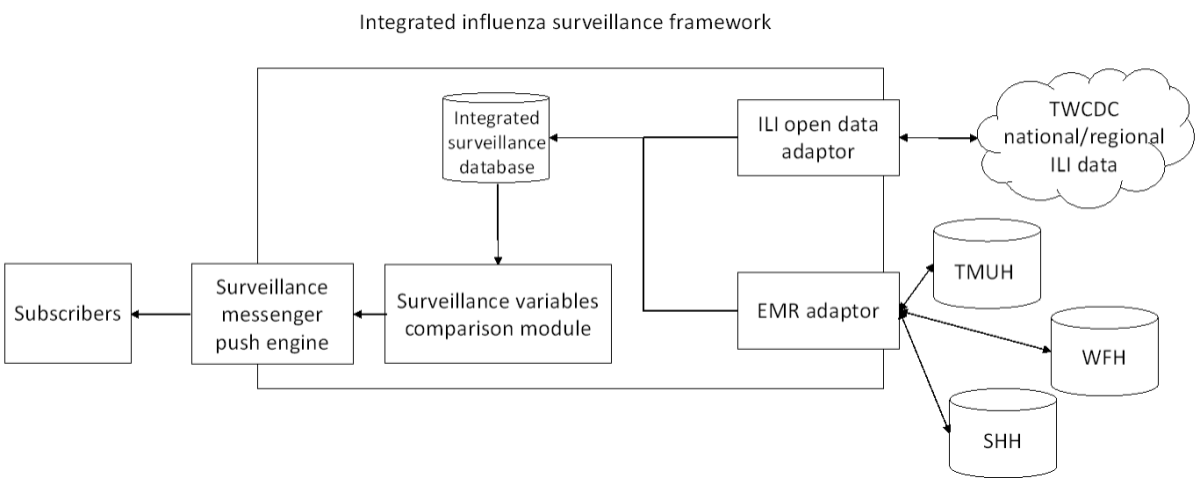
Integrated Influenza Surveillance Framework for influenza epidemic surveillance. ILI: influenza-like illness; EMR: electronic medical record; TWCDC: Taiwan Centers for Disease Control and Prevention; TMUH: Taipei Medical University Hospital; WFH: Wan Fang Hospital; SHH: Shuang Ho Hospital.

We developed an Integrated Influenza Surveillance Framework that included 5 components (Figure 1): ILI Open Data Adapter, EMR Adapter, Integrated Surveillance Database, Surveillance Variables Comparison Module, and Surveillance Messenger Push Engine.

#### Influenza-Like Illness Open Data Adapter

The ILI Open Data Adapter included a Web crawler that can periodically initiate access requests to the TWCDC ILI open data website [32]. The TWCDC ILI data included the year, week number, ILI case counts, and the corresponding areas. Once the ILI Open Data Adapter retrieved the ILI data, these were classified into national (Taiwan)- and regional (Taipei City and New Taipei City)-level data and then deposited into the Integrated Surveillance Database.

#### Electronic Medical Records Adapter

The EMR Adapter created a connection with TMUHcS hospital EMRs and extracted EMRs according to the definition of each surveillance variable. Once the specific surveillance variables were retrieved from the EMRs, these data were stored in the Integrated Surveillance Database.

#### Integrated Surveillance Database

The Integrated Surveillance Database stored national- and regional-level ILI data from TWCDC and specific surveillance variables data from TMUHcS hospital EMRs. These data were then input into the Surveillance Variables Comparison Module for further processing.

#### Surveillance Variables Comparison Module

This module included an automated procedure developed using the Java programming language. It could access surveillance variable data from the Integrated Surveillance Database and conduct Pearson correlation analyses as well as Moving Epidemic Method (MEM) analyses. In this module, the Java programming language invoked the SAS Integrated Object Model [37] to conduct Pearson correlation analyses and rJava [38] to access the R server to perform MEM analysis.

In the initial stage of Surveillance Variables Comparison Module development, Pearson correlation analysis was performed to assess the association between each TMUHcS surveillance variable from the EMRs and the national and regional TWCDC ILI data. After the initial stage, Pearson correlation analysis was not performed at the time of data collection but rather after accumulating the annual data to ascertain that TMUHcS EMRs and TWCDC ILI data reached a significant correlation. For example, once a surveillance variable from TMUHcS EMR data was identified to have a strong correlation (correlation coefficient >.7 and *P* ≤.05) with TWCDC regional and national ILI data, MEM was used to assess whether the daily number of accumulated cases of the surveillance variable surpassed its influenza epidemic threshold. If the value was over the threshold, the Surveillance Variable Comparison Module was made to activate the Surveillance Messenger Push Engine to conduct subsequent tasks.

#### Surveillance Messenger Push Engine

To provide immediate monitoring results to hospital infection control practitioners, the publish-subscribe pattern was adopted in the Surveillance Messenger Push Engine. The publish-subscribe pattern was developed using Amazon Simple Notification Service [39,40] to manage the subscribes and push messages, such as influenza epidemic alert information. Subscribers (eg, hospital infection control practitioners) could subscribe to this messaging service using the hospital's mobile app or the computerized physician order entry (CPOE) system. When the Surveillance Messenger Push Engine was activated by the Surveillance Variables Comparison Module, it sent the alert messages to each subscriber; therefore, the subscribers could receive and check the alert messages via their mobile devices or the CPOE system.

### Variables for Influenza Surveillance

A team of infection control specialists, clinicians, and information technology professionals worked together to identify relevant surveillance variables from EMRs. Unstructured data (ie, data in free-text format) were excluded, and only the structured data including diagnosis codes, medications prescribed, and laboratory tests and results were retained. All of the selected surveillance variables can be automatically extracted from TMUHcS EMRs. In this study, we identified 3 relevant surveillance variables: (1) TMUHcS-ILI, which referred to patients whose diagnosis codes were related to ILI symptoms on the basis of the TWCDC ILI case definition [32], (2) TMUHcS-RITP, which referred to patients whose RIDTs were positive on the basis of ILI diagnosis codes, and (3) TMUHcS-IMU, which referred to patients who were prescribed antiviral drugs for influenza on the basis of ILI diagnosis codes.

Each surveillance variable was independently evaluated for the EMR of each outpatient. For example, if an outpatient’s EMR contained ILI diagnosis codes and a positive RIDT result, both TMUHcS-ILI and TMUHcS-RITP were considered positive. Descriptions of the surveillance variables and their corresponding definitions are summarized in Table 1.

### Moving Epidemic Method

The MEM can be used to define baseline influenza activity from historical data and establish an absolute threshold, above which the weekly infection rates are considered to represent an epidemic period [41-43]. The absolute threshold can be used to define the start and end of the epidemic period. We used MEM to evaluate the predictive power of each surveillance variable for influenza epidemics. In addition, MEM was used to determine the absolute threshold for each surveillance variable and to determine the surveillance variables that reached the epidemic peak the soonest. Moreover, we estimated and compared the absolute threshold and epidemic period for each surveillance variable to determine the most useful variables for early prediction of influenza epidemics.

### Data Collection

We retrospectively collected data from TMUHcS EMRs and TWCDC ILI data (both regional and national) over a 3-year period from October 2014 to September 2017. For TMUHcS EMRs, all outpatients who visited TMUHcS hospitals between October 1, 2014 and September 30, 2017 were included in this study. However, TMUHcS ED patients were excluded in this study because the case definition of the TWCDC ED ILI was different and had a wider range than that of the TWCDC OPD ILI [44]. Moreover, official TWCDC ILI reports to the public were mainly on the basis of the OPD ILI data. Therefore, in this study, we only used TMUHcS OPD EMRs for comparison with TWCDC ILI data. TWCDC ILI data are freely distributed and available from the TWCDC website [32]. Generally, TWCDC announces the latest ILI reports to the public with a time lag of approximately 1 week.

**Table 1:** Influenza surveillance variables and the corresponding data definitions.

Surveillance variable (abbreviation)	Description	Definition
ILI^a^ diagnosis codes [32] (TMUHcS^b^-ILI)	Patients who were diagnosed with ILI-related symptoms	ILI diagnosis codes: (1) ICD-9-CM^c^ codes: 480.xx~487.xx.; (2) ICD-10-CM codes^d^: J09-~J18-, A22-, A37-, B25-, and B44-
Rapid influenza laboratory tests with positive results (TMUHcS-RITP^e^)	Patients who had positive results on rapid influenza tests based on ILI diagnosis codes	Rapid Influenza laboratory tests: positive, + And ILI diagnosis codes: (1) ICD-9-CM codes: 480.xx~487.xx (2) ICD-10-CM codes^d^: J09-~J18-, A22-, A37-, B25-, and B44-
Influenza medication use (TMUHcS-IMU^f^)	Patients who were prescribed with antiviral drugs against influenza based on ILI diagnosis codes	Medications: Tamiflu, Relenza, Rapiacta, and Avigan And ILI diagnosis codes: (1) ICD-9-CM codes: 480.xx-487.xx (2) ICD-10-CM codes^d^: J09- to J18-, A22-, A37-, B25-, and B44-

^a^ILI: influenza-like illness.

^b^TMUHcS: Taipei Medical University Health Care System.

^c^ICD-9-CM: International Classification of Disease, Ninth Revision, Clinical Modification.

^d^Implemented in Taiwan in January 2016.

^e^RITP: rapid influenza laboratory tests with positive results.

^f^IMU: influenza medication use.

### Data Analysis

Data analysis was performed weekly over a 12-month period to determine the week with the highest influenza incidence, according to different surveillance variables. The entire study duration was categorized into 3 periods: Period 1 (October 2014 to September 2015), Period 2 (October 2015 to September 2016), and Period 3 (October 2016 to September 2017).

According to the definitions of each surveillance variable, the number of influenza cases was analyzed to determine the proportion of the corresponding population affected on a weekly basis. To investigate in more detail the comparability of the weekly patterns, we used the locally estimated scatterplot smoothing for weekly data of each surveillance variable [45]. Using the statistical software SAS 9.4 (SAS Institute), Pearson correlation coefficients were calculated to assess the association of each surveillance variable from the EMRs with that from the national and regional TWCDC ILI data. A strong correlation was defined as correlation coefficient of >.7 and statistical significance at a *P* ≤.05. We used MEM to estimate the early predictive power of each surveillance variable. Data analysis was performed in R 3.4.2 (CRAN) [46] using the MEM library version 2.11 [47]. The Joint Institutional Review Board of Taipei Medical University and TMUHcS approved this study.

## Results

### Study Population

From October 1, 2014 to September 30, 2017 we collected a total of 156 weeks of the TWCDC ILI (both regional- and national-level) and TMUHcS EMRs data through the Integrated Influenza Surveillance Framework. Over this 3-year period, the total number of outpatient visits for the national (Taiwan), regional (Taipei Metropolitan), and TMUHcS hospitals were 759,306,494, 214,161,277, and 12,679,529, respectively. Overall, 8,772,125 (1.16%), 1,950,577 (0.91%), 128,378 (1.01%), 3888 (3.03%), and 8463 (6.59%) OPD cases presented with ILI in the national ILI, regional ILI, TMUHcS-ILI, TMUHcS-RITP, and TMUHcS-IMU datasets, respectively. Moreover, of all the TMUHcS-ILI OPD cases, 8094 (6.30%) were advised to undergo RIDTs; of these RIDTs cases, 3888 (48.04%) had positive results. The descriptive statistics for the study population are presented in Multimedia Appendix 1.

### Time Series Trends for Influenza Surveillance Variables

**Figure 2:**
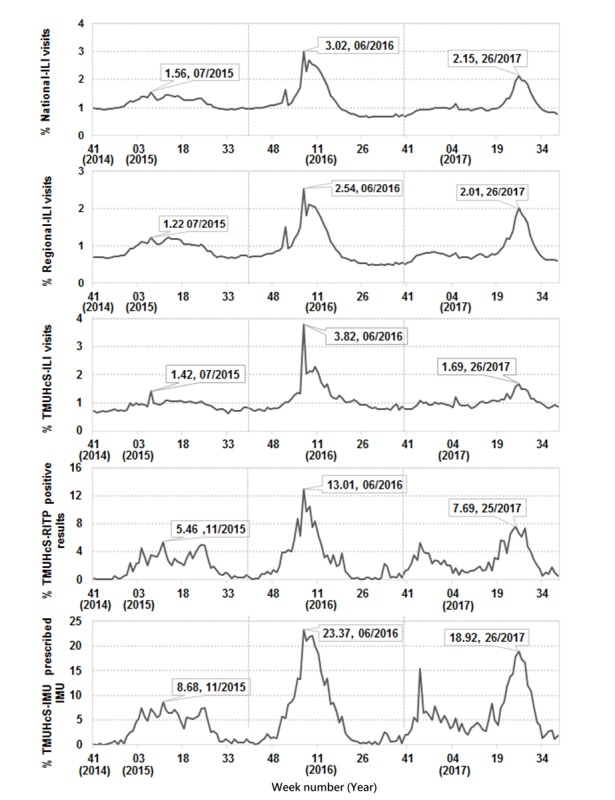
Timeline of influenza-related case proportions based on national influenza-like illness (ILI) data, regional ILI data, and surveillance variables from the electronic medical records of Taipei Medical University Health Care System (TMUHcS; October 2014 to September 2017). IMU: influenza medication use; RITP: rapid influenza laboratory tests with positive results.

The numbers and proportions of cases occurring over a week varied according to each surveillance variable (ie, national ILI, regional ILI, TMUHcS-ILI, TMUHcS-RITP, and TMUHcS- IMU; Figure 2). The highest peak for all surveillance variables occurred during the sixth week of 2016, during which the proportions of influenza-related OPD visits were 3.02%, 2.54%, 3.82%, 13.01%, and 23.37% of the national ILI, regional ILI, TMUHcS-ILI, TMUHcS-RITP, and TMUHcS-IMU data, respectively.

In 2017, the peak number of cases for all surveillance variables was lower than that in 2016. However, all variables reached the peak between the twenty-fifth and twenty-sixth week of 2017, during which the proportions of influenza-related OPD visits were 2.15%, 2.01%, 1.69%, 7.69%, and 18.92% of the national ILI, regional ILI, TMUHcS-ILI, TMUHcS-RITP, and TMUHcS-IMU data, respectively. However, the epidemic peaks identified using each surveillance variable in 2015 involved fewer cases and differed from those reported in the subsequent 2 years. In 2015, the peaks identified using the national ILI, regional ILI, and TMUHcS-ILI data occurred during the seventh week, with influenza-related case proportions of 1.56%, 1.22%, and 1.42%, respectively. In contrast, the epidemic peaks identified using TMUHcS-RITP and TMUHcS-IMU data occurred during the eleventh week, with influenza-related case proportions of 5.46% and 8.68%, respectively.

### Correlation Analysis for Surveillance Variables

Tables 2 and 3 show the Pearson correlation coefficients assessing the relationships between each surveillance variable of TMUHcS EMRs data and those of TWCDC regional and national ILI data. Over the entire study period, the correlation coefficient between the national and regional ILI data was .99 (*P*<.05) and remained constant over the 3-year study period.

Overall, Pearson correlation coefficients between the regional ILI data and each TMUHcS variable ranged from 0.86 to 0.90 (Table 2). All of the surveillance variables showed statistically significant correlation coefficients (*P*<.05) with regional ILI, with TMUHcS-IMU showing the strongest overall correlation (r=.90, *P*<.05). The strongest correlation coefficient of .97 (*P*<.05) was observed between TMUHcS-ILI and regional ILI during period 1 and between TMUHcS-IMU and regional ILI during period 2. Moreover, TMUHcS-RITP was strongly correlated with regional ILI during period 2 (r=.92, *P*<.05).

Similarly, over the entire study period, Pearson correlation coefficients between national ILI and each of the TMUHcS surveillance variables ranged from 0.88 to 0.93 (*P*<.05; Table 3). All variables showed statistically significant correlation coefficients (*P*<.05), whereas TMUHcS-IMU showed the strongest correlation with national ILI (r=.93, *P*<.05). The strongest correlation coefficient of .98 (*P*<.05) was observed between TMUHcS-ILI and national ILI during period 1. TMUHcS-RITP during period 2 (r=0.95, *P*<.05) and TMUHcS-IMU during period 2 (r=0.97, *P*<.05) were strongly correlated with national ILI.

### Early Prediction of Influenza Epidemics

**Figure 3:**
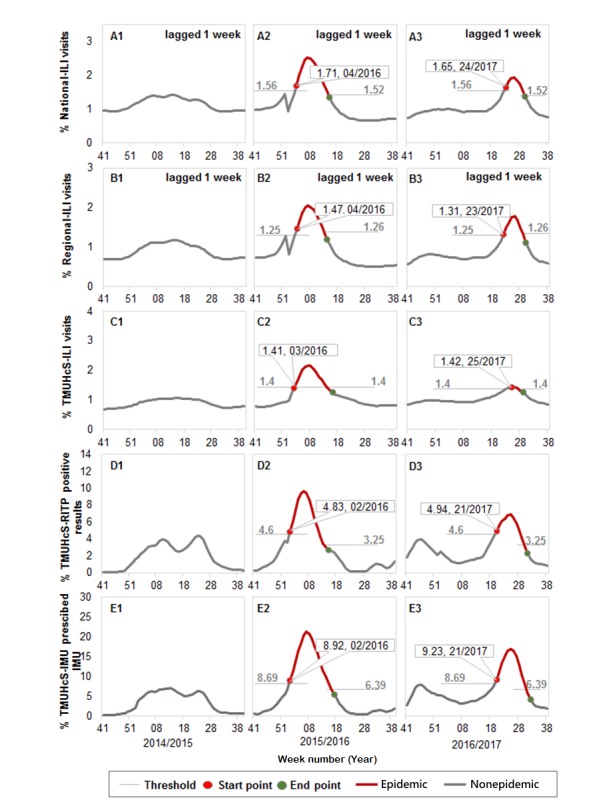
Epidemic thresholds for each surveillance variable (October 2014 to September 2017; locally estimated scatterplot smoothing adjusted). TMUHcS: Taipei Medical University Health Care System; ILI: influenza-like illness; IMU: influenza medication use; RITP: rapid influenza laboratory tests with positive results.

In Figure 3, the absolute thresholds for the 3-year study period were 1.56% to 1.52% for national ILI, 1.25% to 1.26% for regional ILI, 1.4% to 1.4% for TMUHcS-ILI, 4.6% to 3.25% for TMUHcS-RITP, and 8.69% to 6.39% for TMUHcS-IMU. The influenza epidemic threshold was not reached by any of the surveillance variables during period 1. During period 2, all of the surveillance variables surpassed the influenza epidemic threshold. TMUHcS-IMU (E2) and TMUHcS-RITP (D2) reached peak epidemic activity during the second week of 2016, followed by TMUHcS-ILI (C2) during the third week of 2016, then by national ILI (A2) and regional ILI (B2) during the fifth week of 2016 (1-week lag). Therefore, the peak epidemic activity according to the TMUHcS-RITP and TMUHcS-IMU was reached 3 weeks before that according to the national ILI and regional ILI during period 2.

During period 3, the influenza epidemic threshold was surpassed by each surveillance variable. TMUHcS-IMU (E3) and TMUHcS-RITP (D3) reached the peak epidemic activity during the twenty-first week of 2017, followed by regional ILI (B3) during the twenty-fourth week of 2017 (1-week lag) and subsequently by TMUHcS-ILI (C3) during the twenty-fifth week of 2017 and national ILI (A3; 1-week lag). Again, the peak influenza activity according to TMUHcS-RITP and TMUHcS-IMU was observed 3 to 4 weeks before that according to the national ILI and regional ILI during period 3.

The TWCDC ILI reports were in complete agreement with our study results (Figure 2). Moreover, the peak influenza epidemic activity predicted by MEM using TMUHcS-IMU and TMUHcS-RITP occurred 3 to 4 weeks earlier than that using national ILI and regional ILI, both in 2016 and 2017 (Figure 3).

**Table 2:** Pearson correlation coefficients between the Taiwan Center for Disease Control Regional influenza-like illness data and each surveillance variable from the electronic medical records of Taipei Medical University Health Care System (October 2014 to September 2017).

Time period	National ILI^a^	TMUHcS^b^-ILI	TMUHcS-RITP^c^	TMUHcS-IMU^d^
Regional ILI of the entire period (October 2014 to September 2017)	.99	.87	.86	.90
Regional ILI of period 1 (October 2014 to September 2015)	.99	.97	.88	.96
Regional ILI of period 2 (October 2015 to September 2016)	.99	.93	.92	.97
Regional ILI of period 3 (October 2016 to September 2017)	.99	.96	.82	.91

^a^ILI: influenza-like illness.

^b^TMUHcS: Taipei Medical University Health Care System.

^c^RITP: rapid influenza laboratory tests with positive results.

^d^IMU: influenza medication use.

**Table 3:** Pearson correlation coefficients between the Taiwan Center for Disease Control National influenza-like illness data and each surveillance variable from the electronic medical records of Taipei Medical University Health Care System (October 2014 to September 2017).

Time period	Regional ILI^a^	TMUHcS^b^-ILI	TMUHcS-RITP^c^	TMUHcS-IMU^d^
National ILI of the entire period (October 2014 to September 2017)	.99	.88	.90	.93
National ILI of period 1 (October 2014 to September 2015)	.99	.98	.87	.96
National ILI of period 2 (October 2015 to September 2016)	.99	.94	.95	.97
National ILI of period 3 (October 2016 to September 2017)	.99	.97	.88	.96

^a^ILI: influenza-like illness.

^b^TMUHcS: Taipei Medical University Health Care System.

^c^RITP: rapid influenza laboratory tests with positive results.

^d^IMU: influenza medication use.

## Discussion

### Principal Findings

The Integrated Influenza Surveillance Framework described in this study had the scalable ability to periodically integrate the TWCDC ILI open data with EMRs of multiple hospitals. The Surveillance Comparison Module could perform daily comparisons with the value of surveillance variables in this study. When the value of each surveillance variable reached or exceeded the epidemic thresholds, the Surveillance Messenger Push Engine proactively sent the emerging infectious disease surveillance alert to the subscribers (eg, health care facilities or epidemic prevention personnel). As our Surveillance Messenger Push Engine adopted the publish-subscribe pattern, the subscribers could receive the emerging infectious disease surveillance alert immediately through the mobile apps or CPOE systems; this novel approach can provide more efficient and instant infection warning compared with the Web application programming interface call or website of health care facilities or passive checking by the epidemic prevention personnel.

In this study, we identified 3 surveillance variables (TMUHcS-ILI, TMUHcS-RITP, and TMUHcS-IMU) from TMUHcS EMRs that showed a predictive value for influenza epidemics in Taiwan. Each TMUHcS surveillance variable was strongly correlated with regional and national ILI data. Compared with the TWCDC national and regional ILI populations, the TMUHcS population showed ILI case proportions of only 1.46% and 6.58%; however, the ILI case proportions for the national ILI, regional ILI, and TMUHcS-ILI were similar at 1.16%, 0.91%, and 1.01%, respectively. On the basis of the strong correlations and similar ILI case proportions between the 2 study datasets, the 10- to 70-fold smaller sample size of TMUHcS EMR data appeared to provide the same power as did the regional and national ILI data in supporting near-real-time influenza surveillance. These findings represent significant savings in the time and effort required for data collection and analysis.

There is a growing interest in exploring more timely and cost-effective surveillance approaches to enhance the early prediction of infectious disease epidemics [19,23,24,28,48,49]. Unlike previous studies that used only a single surveillance variable or a combination of algorithms to predict seasonal influenza epidemics, our approach was simple, easy to implement, and logically based on physician behaviors. Most importantly, the predictive power of the selected surveillance variables was evaluated using MEM. Of the 3 selected surveillance variables, TMUHcS-RITP and TMUHcS-IMU showed the best predictive power for influenza epidemics and predicted seasonal influenza epidemics 3 to 4 weeks ahead of the TWCDC ILI reports. Furthermore, the types of influenza vaccines recommended by WHO in 2018 might imperfectly match the circulating strains; they were approximately 30% effective against H3N2 viruses [50] but failed to prevent some epidemics. Therefore, the importance of early and accurate predictive ability of our approach is further highlighted for early epidemic control because it provided insights into the mismatches of the influenza virus types for national flu vaccination programs.

Taiwan is located in the subtropical regions of Southeast Asia and has several densely populated urban areas with well-established public transportation networks. There are frequent travelers from Taiwan to regions in which influenza pandemics have originated in the past, such as Hong Kong and mainland China [51]. Therefore, emerging infectious diseases can easily be transmitted from person to person in Taiwan, resulting in the rapid spread of an epidemic. Efficient and real-time monitoring tools for such diseases have become increasingly important. Our approach can be used for predicting influenza epidemics and can be easily extended to other infectious diseases by simply selecting different surveillance variables from EMRs. Accordingly, our approach can play a key role in public health and hospital preparedness, which is the top priority now more than ever.

The peaks of influenza epidemics appear to recur annually, as shown in our study. The influenza season in Taiwan commonly begins around October, peaks after the end of December, and ends in February. We closely examined the different time periods of our study datasets and noticed the following trends. During the 3-year study period, no influenza epidemic was observed during the first year; however, high-intensity influenza activity and an epidemic were observed during the second year, but no obvious influenza activity was noted during the third year (ie, October 2016 to February 2017). However, during the summer of 2017 (June-July), unprecedented levels of influenza activity were observed. Our medical team hypothesized that this delay in the occurrence of influenza epidemic resulted from the unusually warm winter from December 2016 to January 2017. The winter of 2016 to 2017 was the hottest on record, with an average monthly temperature that was 2°C higher than the historical average. This might indicate an association between warm winters and delayed occurrence of influenza epidemics.

Accumulating evidence has shown that EMR systems can facilitate better documentation, communication, and integration of health care data [52]. Accordingly, our results demonstrated that an integrated approach generated a simple, accurate, and timely electronic surveillance tool for predicting influenza epidemics, particularly in densely populated areas and smaller countries. With increased adoption of EMR systems, more meaningful surveillance variables and their corresponding EMRs can be collected and used to support various forms of emerging infectious disease surveillance in the future.

### Conclusions

In this study, we developed an Integrated Influenza Surveillance Framework that had a scalable ability to periodically integrate TWCDC ILI open data with EMRs of multiple hospitals. The system allowed for the automatic monitoring of influenza activity, supporting the early surveillance of influenza epidemics. Using EMRs of multiple hospitals, we identified 3 selected surveillance variables for the early prediction of influenza epidemics. Each surveillance variable was strongly correlated with TWCDC regional and national ILI data. Most importantly, we observed that the predictive power of the 2 selected surveillance variables was 3 to 4 weeks advanced compared with that of the TWCDC ILI reports. Future research should involve further development of this accurate, timely, and cost-effective electronic influenza surveillance tool for the prediction of influenza epidemics as well as the extension of this approach to other infectious diseases in ways that can be particularly relevant to densely populated regions and nations.

## Appendix

Multimedia Appendix 1 Descriptive statistics for the outpatient visits associated with influenza-like illness from October 2014 to September 2017.

## Abbreviations

CDC: Centers for Disease Control and Prevention
CPOE: computerized physician order entry
ED: emergency department
EMR: electronic medical record
ILI: influenza-like illness
IMU: influenza medication use
MEM: Moving Epidemic Method
NHI: National Health Insurance
OPD: outpatient department
RIDT: rapid influenza diagnostic test
RITP: rapid influenza laboratory tests with positive results
RT-PCR: reverse transcription polymerase chain reaction
TMUHcS: Taipei Medical University Health Care System
TWCDC: Taiwan Centers for Disease Control and Prevention
WHO: World Health Organization
