# Author Contribution Correction: An Integrated Influenza Surveillance Framework Based on National Influenza-Like Illness Incidence and Multiple Hospital Electronic Medical Records for Early Prediction of Influenza Epidemics: Design and Evaluation

**DOI:** 10.2196/13699

**Published:** 2019-03-12

**Authors:** Cheng-Yi Yang, Ray-Jade Chen, Wan-Lin Chou, Yuarn-Jang Lee, Yu-Sheng Lo

**Affiliations:** 1 Graduate Institute of Biomedical Informatics Taipei Medical University Taipei Taiwan; 2 Department of Surgery School of Medicine College of Medicine, Taipei Medical University Taipei Taiwan; 3 Taipei Medical University Hospital Taipei Taiwan; 4 Division of Infectious Disease Department of Internal Medicine Taipei Medical University Hospital Taipei Taiwan

The authors of “An Integrated Influenza Surveillance Framework Based on National Influenza-Like Illness Incidence and Multiple Hospital Electronic Medical Records for Early Prediction of Influenza Epidemics: Design and Evaluation” (J Med Internet Res 2019;21(2):e12341) inadvertently marked Yu-Sheng Lo as an equal contributor when that designation should have only been applied to Cheng-Yi Yang and Ray-Jade Chen. The asterisk denoting equal contribution has now been removed from Yu-Sheng Lo.

The correction will appear in the online version of the paper on the JMIR website on March 11, 2019, together with the publication of this correction notice. Because this was made after submission to PubMed, PubMed Central, and other full-text repositories, the corrected article also has been resubmitted to those repositories.

