# Supplementary material for: An Integrated Influenza Surveillance Framework Based on National Influenza-Like Illness Incidence and Multiple Hospital Electronic Medical Records for Early Prediction of Influenza Epidemics: Design and Evaluation
Source: J Med Internet Res. 2019 Feb 1;21(2):e12341. doi: 10.2196/12341 (PMC6376337; doi:10.2196/12341)
Supplement: Multimedia Appendix 1 [file jmir_v21i2e12341_app1.docx]

Appendix 1. Descriptive statistics for the outpatient visits associated with influenza-like illness from October 2014 to September 2017.

| Time period | National ILI | | Regional ILI^a^ | | TMUHcS-ILI | | TMUHcS-RITP | | TMUHcS-IMU |
| --- | --- | --- | --- | --- | --- | --- | --- | --- | --- |
|  | Total visits^1^ | ILI visits  (% of total) | Total visits^2^ | ILI visits  (% of total) | Total visits^3^ | ILI visits^4^  (% of total) | Total RIDTs^5^  (% of total ILI) | Positive results^6^  (% of total ILI; % of total RIDTs) | Prescribed IMU  (% of total ILI) |
| Entire period | 759,306,494 | 8,772,125  (1.16) | 214,161,277 | 1,950,577  (0.91) | 12,679,529 | 128,378  (1.01) | 8094  (6.30) | 3888  (3.03; 48.04) | 8463  (6.59) |
| Period 1 | 251,889,438 | 2,918,758  (1.16) | 70,772,692 | 634,844  (0.90) | 4,170,470 | 36,594  (0.88) | 1716  (4.69) | 780  (2.13; 45.45) | 1367  (3.74) |
| Period 2 | 255,647,129 | 3,111,849  (1.22) | 71,956,914 | 682,459  (0.95) | 4,198,597 | 46,892  (1.12) | 3315  (7.07) | 1656  (3.53; 49.95) | 3787  (8.08) |
| Period 3 | 251,769,927 | 2,741,518  (1.09) | 71,431,671 | 633,274  (0.90) | 4,310,462 | 44,892  (1.04) | 3063  (6.82) | 1452  (3.23; 47.40) | 3309  (7.37) |
| ^a^Regional areas included Taipei City and New Taipei City.  ^1^Total national outpatient visits.  ^2^Total regional outpatient visits.  ^3^Total TMUHcS outpatient visits.  ^4^Total TMUHcS outpatient ILI visits.  ^5^Total RIDTs number of total TMUHcS outpatient ILI visits.  ^6^Total positive results of total RIDTs number  ILI: Influenza-like illness.  IMU: Influenza medication use.  RIDTs: Rapid influenza diagnostic tests.  RITP: Rapid influenza laboratory tests with positive results.  TMUHcS: Taipei Medical University Health Care System. | | | | | | | | | |
